# Four-year trends in adiposity and its association with hypertension in serial groups of young adult university students in urban Cameroon: a time-series study

**DOI:** 10.1186/s12889-017-4449-7

**Published:** 2017-05-23

**Authors:** Simeon-Pierre Choukem, André-Pascal Kengne, Maxime-Leolein Nguefack, Yannick Mboue-Djieka, Daniel Nebongo, Jackson T Guimezap, Jean Claude Mbanya

**Affiliations:** 1 Department of Internal Medicine and Pediatrics, Faculty of Health Sciences, University of Buea, P.O. Box 4856, Douala, Buea, Cameroon; 2 Health and Human Development (2HD) Research Network, Douala, Cameroon; 3 Diabetes and Endocrine Unit, Department of Internal Medicine, Douala General Hospital, Douala, Cameroon; 4 South African Medical Research Council, University of Cape Town, Cape Town, South Africa; 5 Higher Institute of Health Sciences, Université des Montagnes, Bangangte, Cameroon; 6 University of Montreal, Montreal, QC Canada; 7 National Center of Obesity, Diabetes and Endocrinology Yaoundé Central Hospital, Yaoundé, Cameroon; 8 Laboratory of molecular and metabolic medicine, Biotechnology Center, University of Yaoundé 1, Yaoundé, Cameroon; 9 Department of Internal Medicine and Subspecialties, Faculty of Medicine and Biomedical Sciences, University of Yaoundé 1, Yaoundé, Cameroon

**Keywords:** Overweight, Obesity, Hypertension, Trends, Association, Cameroon

## Abstract

**Background:**

Obesity is a major risk factor for non-communicable diseases (NCDs) and is growing rapidly globally including in sub-Saharan Africa (SSA). We aimed to assess the trend in adiposity markers in Cameroonian university students, and investigated their associations with hypertension.

**Methods:**

From 2009 to 2012, we annually measured weight, height, blood pressure, waist (WC) and hip circumferences, and calculated the body mass index (BMI) and other indices of adiposity in consecutive students aged 18 years or above, during their registration. Time-trends in prevalence of overweight and obesity were estimated, and their associations with prevalent hypertension investigated.

**Results:**

Among the 2726 participants, the overall prevalence of obesity, overweight and obesity combined, and hypertension was 3.5%, 21.0% and 6.3% respectively. From 2009 to 2012, the prevalence of overweight and obesity increased in men only, from 13.1% to 20.9% (p-trend = 0.002), whereas prevalent abdominal obesity increased in women only, from 6.5% to 11.7% (p-trend = 0.027). The BMI and the WC were independent predictors of hypertension; each kg/m^2^ higher BMI was associated with 11% higher odds of hypertension, and each centimeter higher WC was associated with 9% higher odds of hypertension.

**Conclusion:**

Our results show that overweight and obesity are rapidly increasing in this population of young sub-Saharan African adults, and are contributing to an increasing burden of hypertension.

## Background

Non-communicable diseases (NCDs) such as cardiovascular diseases, diabetes, hypertension and cancers are growing rapidly globally including in sub-Saharan Africa (SSA) [1–3]. The most effective strategy to tackle these NCDs is to prevent them by targeting their risk factors and their upstream determinants as early as possible in the life course. Obesity is a major risk factor for NCDs and is increasing rapidly. The latest Global burden of obesity report shows that the global prevalence of overweight/obesity increased between 1980 and 2013 from 28.8 to 36.9% in men, and from 29.8 to 38.0% in women [4]. Across West African countries comprising Cameroon, estimations show that the prevalence of combined overweight and obesity in 2013 was 32.6% in men and 34.5% in women aged 20 years and above, with the corresponding prevalence in Cameroon being 40.4 and 50.7% [4]. Moreover, this report also shows that successive cohort in both developed and developing countries seemed to be gaining weight at all ages, with most rapid gains between the ages of 20 and 40 years [4].

Developed countries have used observational studies to establish the time-trends in obesity in order to inform preventive measures. The ObEpi survey in France shows that the prevalence of overweight and obesity in adults ≥18 years has risen from 46% in 2009 to 47% in 2012 [5]. In the sub-population aged 18 to 34 years, the prevalence of obesity alone increased from 14.4% in 2009 to 16.3% in 2012. Concomitantly the waist circumference increased from 94.8 cm to 95.1 cm in males and 85.5 cm to 86.5 cm in females; the ObEpi survey also showed that compared with people with BMI < 25 kg/m2, people with overweight or obesity were 2.3 fold and 3.6 fold more at risk to have hypertension, respectively [5].

These time-trends have been less well assessed in SSA, and data in the global burden of obesity or other non-communicable diseases are often based on extrapolations. Whether such a rise in overweight and obesity within a short period of time would be seen in SSA is not known, as well as its potential consequences on obesity-related conditions such as hypertension. The aim of this study was to assess the trends in adiposity parameters in Cameroonian university students, and to investigate their potential associations with prevalent hypertension.

## Methods

### Study design, participants and setting

We conducted a time-series study in a private university institute in Douala, the most populous city of Cameroon with an estimated population of 2.45 million inhabitants in 2011. Participants were students who presented for their first registration in the institution. They were either new graduates from high school or were coming from other universities. All students aged 18 years or above were included. Those who refused to provide a written informed consent were excluded. Data collection took place during compulsory health assessment as part of the requirements for registration at the institution. The health package provided to each student included characterization of the adiposity status, measurement of blood pressure (BP) and education on lifestyle measures against obesity, diabetes and hypertension. Students were asked to give their written consent to have their data exploited for research purposes. Pregnant women were excluded from the analysis. The study was approved by the Institutional Ethics Committee of *Université des Montagnes*.

### Data collection

Data were collected each year for four consecutive years by two doctors, over a period of four weeks (mid-October to mid-November) in 2009, 2010, 2011 and 2012. Doctors collecting the data had been trained for that purpose and used validated methods. Data collected included: the BP, weight (to the nearest 100 g), height (to the nearest 0.5 cm), waist (WC) and hip (HC) circumferences to the nearest 0.5 cm. The BP was measured in the morning after 5 min of rest in a sitting position, using an OMRON M3® (OMRON HEALTHCARE Co., Kyoto, Japan) with an appropriate cuff. The weight (in kg) was measured with a Camry® scale that was recalibrated to zero before each measurement, while participants wearing light clothes and without shoes. The waist circumference was taken at mid distance between the iliac crest, and the hip circumference at the level of the great trochanters. The body mass index (BMI) was calculated as the weight in kg divided by the square of the height in meter (kg/m^2^). Other secondary variables included waist-to-hip ratio (WHR) and waist-to-height ratio (WHtR) were calculated by simple division.

Overweight was defined as BMI between 25 and 29.9 kg/m^2^, and obesity as BMI ≥ 30 kg/m2. Hypertension was defined based on a single measurement, as systolic blood pressure ≥ 140 mmHg and/or diastolic blood pressure ≥ 90 mmHg or current antihypertensive treatment.

### Statistical analysis

Data were analysed with the use of SAS/STAT® version 9.1 for Windows (SAS Institute Inc., Cary, NC, USA). Results are presented as counts and percentages, mean and standard deviation (SD). Differences between men and women and across years of study were investigated via chi square tests and equivalents for qualitative variables. Linearity in the trend across years was assessed with the Cochran-Armitage trend test, while the analysis of the variance (ANOVA) and equivalents were used for quantitative variables. Logistic regressions models were used to investigate the baseline characteristics associated with prevalent hypertension. Basic regression models were adjusted for age and sex, while the extended multivariable models also comprised significant predictors in basic models, based on a threshold for significance of *p* ≤ 0.10. A *p*-value <0.05 was used to characterize statistically significant results.

## Results

### General characteristics of participants

The total study population was 2726. The annually included number of participants represented 20.5% of the total population in 2009, 17.2% in 2010, 25.3% in 2011, and 37.0% in 2012 (Table 1). Participant’s age ranged from 18 to 38.7 years, with a mean (SD) of 21.8 (2.4) years, and was similar across years of study (*p* = 0.271). Gender distribution significantly differed in proportion between years (Table 1). The overall prevalence of obesity, overweight and obesity combined, and hypertension were 3.5%, 21.0 and 6.3% respectively (Table 2). The overall prevalence of abdominal obesity was 0.5% in men and 7.8% in women.

**Table 1:** Profile of participants overall and across years of the study

Variables	Overall	2009	2010	2011	2012	*p*-value	p-trend
N (%)	2726 (100)	559 (20.5)	469 (17.2)	690 (25.3)	1008 (37.0)		
Men, n (%)	1893 (69.4)	389 (69.6)	300 (64.0)	504 (73.0)	700 (69.4)	0.013	0.383
Mean age, years	21.8 (2.4)	21.8 (2.4)	21.7 (2.4)	21.9 (2.5)	21.7 (2.4)	0.271	0.850
Pulse, beats per min	77 (13)	76 (13)	79 (13)	75 (13)	77 (14)	<0.0001	0.314
Weight, kg	67.8 (10.6)	67.1 (10.2)	67.0 (10.9)	67.6 (10.0)	68.7 (10.9)	0.177	0.005
Height, cm	171 (9)	171 (9)	171 (9)	171 (8)	171 (9)	0.687	0.232
Hip circumference, cm	96 (7)	96 (9)	96 (8)	94 (7)	96 (8)	<0.0001	<0.0001
Men							
N (%)	1893 (100)	389 (20.6)	300 (15.9)	504 (26.6)	700 (36.9)		
Mean age, years	21.9 (2.5)	22.0 (2.5)	21.9 (2.5)	22.1 (2.6)	21.8 (2.4)	0.166	0.420
Pulse, beats per min	74 (13)	74 (12)	77 (13)	73 (12)	74 (13)	0.0004	0.355
Weight, kg	69.8 (9.4)	68.6 (8.9)	69.9 (9.7)	69.3 (9.3)	70.7 (9.6)	0.002	0.288
Height, cm	174.4 (7.0)	174 (7)	175 (7)	174.2 (6.6)	174.5 (7.0)	0.130	0.618
Hip circumference, cm	94.4 (6.9)	94.5 (7.5)	95.4 (6.6)	93.2 (5.9)	94.8 (7.1)	<0.0001	0.004
Women							
N (%)	833 (100)	170 (20.4)	169 (20.3)	186 (22.3)	308 (37.0)		
Mean age, years	21.4 (2.3)	21.6 (2.3)	21.4 (2.0)	21.3 (2.1)	21.4 (2.5)	0.626	0.186
Pulse, beats per min	83 (13)	82 (12)	83 (12)	82 (14)	85 (13)	0.098	0.818
Weight, kg	63.4 (11.7)	63.6 (12.1)	61.9 (11.1)	63.2 (10.4)	64.2 (12.4)	0.236	0.767
Height, cm	163.7 (7.2)	163.7 (7.2)	163.9 (6.0)	164.0 (7.2)	163.3 (7.7)	0.773	0.769
Hip circumference, cm	98.8 (9.4)	100.6 (9.9)	98.3 (9.0)	97.6 (8.2)	98.8 (10.0)	0.018	0.002

**Table 2:** Trends in adiposity and blood pressure parameters in the whole population

Variables	Overall	2009	2010	2011	2012	*p*-value	*p*-trend	p year*gender
N (%)	2726 (100)	559 (20.5)	469 (17.2)	690 (25.3)	1008 (37.0)			
SBP, mmHg)	118 (12)	119 (11)	118 (11)	117 (13)	118 (13)	0.008	0.0007	0.695
DBP mmHg	70 (10)	72 (9)	72 (10)	69 (10)	70 (10)	<0.0001	<0.0001	0.841
Hypertension, n (%)	172 (6.3)	33 (5.9)	26 (5.6)	36 (5.2)	77 (7.6)	0.170	0.141	0.430
BMI, kg/m^2^	23.1 (3.3)	23.0 (3.3)	22.9 (3.2)	23.0 (2.9)	23.5 (3.5)	0.0009	0.965	0.306
Obesity, n (%)	95 (3.5)	17 (3.0)	14 (3.0)	15 (2.2)	49 (4.9)	0.019	0.054	0.500
Overweight and obesity, n (%)	571 (21.0)	95 (17.0)	96 (20.5)	131 (19.0)	249 (24.7)	0.001	0.0005	0.832
Waist circumference, cm	76 (7)	76 (7)	75 (7)	76 (6)	76 (8)	0.095	0.975	0.170
Abdominal obesity, n (%)	73 (2.7)	12 (2.2)	10 (2.3)	11 (1.6)	40 (4.0)	0.016	0.032	0.601
Hip circumference, cm	96 (7)	96 (9)	96 (8)	94 (7)	96 (8)	<0.0001	<0.0001	0.021
WHR, cm	0.79 (0.05)	0.79 (0.05)	0.78 (0.05)	0.81 (0.04)	0.79 (0.06)	<0.0001	<0.0001	<0.0001
WHtR, cm	0.44 (0.05)	0.44 (0.04)	0.44 (0.04)	0.44 (0.04)	0.44 (0.05)	0.177	0.513	0.096

*p-value adjusted for year and gender; Data are presented as mean (standard deviation), unless indicated otherwise; *DBP* diastolic blood pressure, *SBP* systolic blood pressure, *WHR*: waist-to-hip ratio, *WHtR* waist-to-height ratio

### Trends in measures of adiposity

Over four years in the whole population, the prevalence of overweight and obesity combined, abdominal obesity, and the mean WHR rose significantly, whereas the mean waist circumference and WHtR ratio remained stable (Table 2). In men, there was a linear rise in the prevalence of overweight/obesity and the mean WHR, but not in the prevalence of abdominal obesity; in women, the prevalence of abdominal obesity and WHR rose linearly, but not the prevalence of overweight/obesity (Table 3).

**Table 3:** Trends in adiposity and blood pressure parameters by gender

Variables	Overall	2009	2010	2011	2012	*p*-value	p-trend
Men, N (%)	1893 (100)	389 (20.6)	300 (15.9)	504 (26.6)	700 (36.9)		
SBP, mmHg	120 (12)	121 (10)	120 (11)	118 (12)	120 (13)	0.003	0.0002
DBP, mmHg	70 (10)	72 (9)	72 (10)	69 (10)	70 (10)	<0.0001	<0.0001
Hypertension, n (%)	133 (7.0)	22 (5.7)	20 (6.7)	30 (5.9)	61 (8.7)	0.160	0.063
BMI, kg/m^2^	22.9 (2.8)	22.7 (2.9)	22.8 (2.8)	22.8 (2.6)	23.2 (2.9)	0.007	0.501
Obesity, n (%)	39 (2.1)	6 (1.5)	7 (2.3)	8 (1.6)	18 (2.6)	0.551	0.336
Overweight and obesity, n (%)	332 (17.5)	51 (13.1)	51 (17.0)	84 (16.7)	146 (20.9)	0.012	0.002
Waist circumference, cm	76.1 (6.5)	76.5 (5.8)	75.9 (6.3)	76.3 (5.6)	75.8 (7.4)	0.320	0.639
Abdominal obesity, n (%)	9 (0.5)	1 (0.3)	1 (0.3)	3 (0.6)	4 (0.6)	0.843	0.429
Hip circumference, cm	94.4 (6.9)	94.5 (7.5)	95.4 (6.6)	93.2 (5.9)	94.8 (7.1)	<0.0001	0.004
WHR	0.81 (0.05)	0.81 (0.04)	0.79 (0.04)	0.82 (0.04)	0.80 (0.06)	<0.0001	0.011
WHtR	0.44 (0.04)	0.44 (0.04)	0.43 (0.04)	0.44 (0.03)	0.43 (0.04)	0.065	0.424
Women, N (%)	833 (100)	170 (20.4)	169 (20.3)	186 (22.3)	308 (37.0)		
SBP, mmHg	113 (12)	114 (11)	114 (11)	113 (14)	113 (13)	0.565	0.241
DBP, mmHg	70 (10)	71 (10)	72 (9)	69 (10)	70 (10)	0.007	0.009
Hypertension, n (%)	39 (4.7)	11 (6.5)	6 (3.6)	6 (3.2)	16 (5.2)	0.426	0.700
BMI, kg/m^2^	23.7 (4.1)	23.7 (4.1)	23.0 (3.9)	23.5 (3.6)	24.1 (4.6)	0.057	0.675
Obesity, n (%)	56 (6.7)	11 (6.5)	7 (4.2)	7 (3.8)	31 (10.1)	0.020	0.064
Overweight and obesity, n (%)	239 (28.7)	44 (25.9)	45 (26.8)	47 (25.3)	103 (33.4)	0.144	0.068
Waist circumference, cm	75.1 (8.8)	75.2 (8.8)	73.7 (8.2)	75.5 (7.6)	75.6 (9.7)	0.149	0.709
Abdominal obesity, n (%)	64 (7.8)	11 (6.5)	9 (5.6)	8 (4.3)	36 (11.7)	0.011	0.027
Hip circumference, cm	98.8 (9.4)	100.6 (9.9)	98.3 (9.0)	97.6 (8.2)	98.8 (10.0)	0.018	0.002
WHR	0.76 (0.05)	0.75 (0.05)	0.75 (0.05)	0.77 (0.05)	0.76 (0.05)	<0.0001	<0.0001
WHtR	0.46 (0.05)	0.46 (0.05)	0.45 (0.05)	0.46 (0.05)	0.46 (0.06)	0.108	0.764

Data are presented as mean (standard deviation), unless indicated otherwise; *DBP* diastolic blood pressure, *SBP*, systolic blood pressure, *WHR*, waist-to-hip ratio, *WHtR*, waist-to-height ratio

### Trends in blood pressure

Systolic blood pressure fluctuated by 3 mmHg in men with a decreasing trend whereas it remained stable in women (Table 3). Diastolic blood pressure fluctuated by 3 mmHg in both genders, with a decreasing trend (Table 3). The prevalence of hypertension remained stable over four years in both genders (Table 3).

### Adiposity variables and prediction of hypertension

In the basic model, all parameters of adiposity were significant predictors of prevalent hypertension, except the WHR (Table 4). In the multivariate models, the BMI and the WC were independent predictors of hypertension; each 1 kg/m^2^ increase in BMI was associated with 11% increase in the prevalence of hypertension, and each 1 cm increase in WC was associated with 9% rise in the prevalence of hypertension (Table 4).

**Table 4:** Age, sex and year of study adjusted predictors of hypertension from logistic regression models

Variable	Basic models		Multivariable models	
	OR (95% CI)	*p*-value	aOR (95% CI)	*p*-value
Age, per year	1.00 (0.94–1.07)	0.918	0.99 (0.93–1.06)	0.770
Sex (men)	1.52 (1.05–2.19)	0.027	1.14 (0.73–1.78)	0.559
Year		0.163		0.300
2009	1 (reference)		1 (reference)	
2010	0.92 (0.54–1.59)		0.89 (0.52–1.55)	
2011	0.86 (0.53–1.39)		0.84 (0.51–1.37)	
2012	1.31 (0.86–1.99)		1.21 (0.79–1.86)	
BMI (per kg/m2)	1.10 (1.06–1.14)	<0.0001	1.11 (1.02–1.20)	0.013
Obesity	2.74 (1.47–5.12)	0.0015		
Obesity or overweight	2.03 (1.45–2.86)	<0.0001		
Waist circumference (per cm)	1.04 (1.03–1.06)	<0.0001	1.09 (1.04–1.15)	0.0004
Abdominal obesity	4.30 (2.15–8.63)	<0.0001		
Hip circumference	1.05 (1.03–1.07)	<0.0001		
WHR	1.64 (0.08–33.09)	0.747		

*CI*, confidence interval, *aOR*, adjusted odd ratio, *WHR*, waist-to-hip ratio, *BMI*, body mass index

## Discussion

Over a period of four years in young adults aged 18 to 39 years in urban Cameroon, our data show a rise in the prevalence of combined overweight and obesity in men from 13.9% in 2009 to 20.1% in 2012, and a rise in the prevalence of abdominal obesity in women from 6.5% in 2009 to 11.7% in 2012. We also show that the BMI and the WC are independent predictors of prevalent hypertension in this population.

Reports on adults above 20 years of age in SSA over the past decades have shown high burden of overweight, obesity and hypertension [6–8], as well as a worsening of obesity burden over time [9]. As the central component of the metabolic syndrome, abdominal obesity tends to aggregate with hypertension and other cardiometabolic risk factors, and is also highly prevalent in SSA [10].

Preparedness to tackle obesity requires data on its burden in younger age groups (where interventions are more likely to yield benefits), as well as data on trends. The Global burden of obesity report shows that successive cohort from 1980 to 2013 in both developed and developing countries tend to gain weight at all ages, with most rapid weight gains occurring between the ages of 20 and 40 years [4]. To that end, developed countries have assessed the trends. In the age group between 20 and 39 years in the USA, the prevalence of obesity alone increase from 23.7% in 1999–2000 to 27.5% in 2007–2008 in men and from 28.4 to 34.0% in women during the same periods, respectively [11]. In France, data from the ObEpi survey show that the prevalence of overweight and obesity combined in adults ≥18 years rose from 46% in 2009 to 47% in 2012 [5]. Concomitantly the waist circumference increased from 94.8 in 2009 cm to 95.1 in 2012 cm in males and 85.5 cm to 86.5 cm in females. In the sub-population aged 18 to 34 years, the prevalence of obesity alone increased from 14.4% in 2009 to 16.3% in 2012.

In SSA, very few studies have focused on these younger adult age groups or have evaluated the time-trends. Data collected in 2004 in Uganda report overall prevalence of 10.4 and 2.3% of overweight and obesity respectively, in young adults aged 18 to 30 years [12]. Unhealthy diet and sedentary lifestyle that accompany the process of epidemiologic transition are the likely explanations for these trends in both developed and developing countries [1].

Early descriptions on the clustering of obesity with hypertension and other cardiovascular risk factors in children, adolescents and young adults emerged in the 1980s in the USA [13]. We found in our study that the BMI and the WC were strong predictors of prevalent hypertension; each 1 kg/m^2^ increase in BMI was associated with 11% increase in the prevalence of hypertension, and each 1 cm increase in WC was associated with 9% rise in the prevalence of hypertension. In the ObEpi survey in France, people with overweight or obesity were respectively 2.3 times and 3.6 more likely to have hypertension compared with people with BMI < 25 kg/m2 [5]. In 1368 adults urban dwellers aged 18 to 88 years in Nigeria with a prevalence of overweight, obesity and hypertension of 32.7%, 22.2 and 33.3%, Amira et al. showed that overweight and obesity were associated with 1.45 and 2.59 odds of prevalent hypertension [14]. In 600 university students aged between 18 and 24 years in Bengal, India, the prevalence of hypertension and combined overweight and obesity were 13 and 35.5% respectively; in this young population, overweight and obesity were associated with higher rates of hypertension [15]. Overall, the results of our study are congruent with observations made elsewhere in developing and developed countries.

Many potential mechanisms have been proposed to explain the role of obesity in the development of hypertension. These include amongst others: activation of the sympathetic nervous system, sodium retention as a results of increased renal tubular reabsorption, and increased renin-angiotensin-aldosterone system activity [16].

We acknowledge the following potential limitations of our study: it took place in a single centre, and participants were not randomly selected. The results may not therefore apply to the general population of young adults. However, these results indicate a clear picture of the burden of overweight and obesity in a young adults population of Cameroon and their role as major risk factor for hypertension; further, they provide trends that are close to those reported in developed countries, and which will be used as a basis for projections. We also could not measure the blood pressure twice as recommended. Lastly, we lack data on some important cardiovascular risk factors we could account for in logistic regressions such as physical activity, diet and sodium intake in particular, and family history of hypertension.

## Conclusion

In this population of young adults of Cameroon, overweight and obesity in men and abdominal obesity in women increase in burden with time. This increase appears as early driving forces for hypertension in this population, and should be targeted early in life so as to curb the increasing burden of hypertension. These results forecast a worsening of cardiovascular diseases especially in women, and call for more aggressive prevention policy against obesity in Cameroon.

## Abbreviations

BMI: Body mass index
BP: Blood pressure
HC: Hip circumference
NCDs: Non-communicable diseases
SSA: Sub Saharan Africa
WC: Waist circumference
WHR: Waist-to-hip ratio
WHtR: Waist-to-height ratio
